# Phosphorylation of TOPK at Y74, Y272 by Src increases the stability of TOPK and promotes tumorigenesis of colon

**DOI:** 10.18632/oncotarget.8231

**Published:** 2016-03-21

**Authors:** Juanjuan Xiao, Qiuhong Duan, Zhe Wang, Wei Yan, Huimin Sun, Peipei Xue, Xiaoming Fan, Xiaoyu Zeng, Juan Chen, Chen Shao, Feng Zhu

**Affiliations:** ^1^ Department of Biochemistry and Molecular Biology, School of Basic Medicine, Huazhong University of Science and Technology, Wuhan, Hubei, 430030, PR China; ^2^ Department of Urology, Xijing Hospital, the Fourth Military Medical University, Xi'an, Shaanxi, 710032, PR China; ^3^ Department of Pathology, Xijing Hospital, the Fourth Military Medical University, Xi'an, Shaanxi, 710032, PR China; ^4^ Laboratory for Translational Oncology Basic Medicine College, Hubei University of Science and Technology, Xianning, Hubei, 437100, PR China

**Keywords:** TOPK, Src, colon cancer, tumorigenesis, stability

## Abstract

T-LAK cell-originated protein kinase (TOPK), a serine/threonine protein kinase, is highly expressed in a variety of tumors and associated with a poor prognosis of human malignancies. However, the activation mechanism of TOPK is still unrevealed. Herein, first we found that Src directly bound with and phosphorylated TOPK at Y74 and Y272 *in vitro*. Anti-phospho-TOPK at Y74 was prepared, the endogenous phosphorylation of TOPK at Y74 was detected in colon cancer cells, and the phosphorylation was inhibited in cells expressing low levels of Src. Subsequently, we stably transfected Y74 and Y272 double mutated TOPK (TOPK-FF) into JB6 or SW480 cells, and observed that both the anchorage-independent growth ability and tumorigenesis of TOPK-FF cells were suppressed compared with those of wild type TOPK (TOPK-WT) *ex vivo* and *in vivo.* The phosphorylation level of TOPK substrate, Histone H3 at Ser10 also decreased dramatically *ex vivo* or *in vivo*. Moreover, we showed that Src could inhibit the ubiquitination of TOPK. Transiently expressed TOPK-WT was more stable than TOPK-FF in pause and chase experiment. Endogenous TOPK was more stable in Src wild type (Src^+/+^) MEFs than in Src knockout (Src^−/−^). Taken together, our results indicate that Src is a novel upstream kinase of TOPK. The phosphorylation of TOPK at Y74 and Y272 by Src increases the stability and activity of TOPK, and promotes the tumorigenesis of colon cancer. It may provide opportunities for TOPK based prognosis and targeted therapy for colon cancer patients.

## INTRODUCTION

TOPK (T-LAK cell-originated protein kinase) is initially identified as a MAPKK-like protein kinase from lymphokine-activated killer T cell and it is also known as PBK (PDZ-binding kinase) linking with the PDZ2domain of tumor suppressor protein hDlg [1, 2]. TOPK is highly expressed in a variety of tumors including breast cancer, colorectal cancer (CRC) and melanoma, and involves in regulating several cell functions such as tumor cell cycle progression [3–6], transformation [7, 8], proliferation [9] and apoptosis [10, 11]. More and more published reports have suggested that elevated levels of TOPK may be associated with the tumorigenesis, metastasis and poor prognosis of cancers [12–17].

Colon cancer is one of the most prevalent cancers worldwide, as well as one of the leading causes of cancer death. American Cancer Society reported that 1 658 370 new cases of cancer would be diagnosed in the United States in 2015 and about 8% cases would be CRC [18]. In previous study, we found that there existed a positive feedback loop between TOPK and ERK2 (extracellular signal-regulated kinase 2), and therefore promoted tumorigenesis in colon cancer *in vitro* and *in vivo* [7]. Moreover, TOPK is a valuable prognostic marker in patients with sporadic CRC, and 30-40% of CRC patients may benefit from the inhibition of TOPK [17]. Deschoolmeester *et al* also reported that TOPK may be a biomarker in prognosis and a therapeutic target in CRC [19]. TOPK is very important in colon cancer. However, only a few kinases including hDlg [2], Cdk1 [2, 3], ERK2 and p38 [7] are known to phosphorylate TOPK at Thr9, and the mechanism of phosphorylation of TOPK at Thr9 have not yet been identified. Therefore, to find out new upstream kinases and other phosphorylation sites would make the progress of clinical application in TOPK field.

Src is the first transforming protein with tyrosine kinase activity discovered and isolated [20]. It can activate multiple signaling pathways, including the PI3K/Akt, MAPK, Stat3, IL-8, VEGF, and cytoskeletal-formation pathways to regulate cellular functions [21]. Src activity increases in 80% of colon cancer patients [22], and the activation of Src can stimulate Ras-Raf-MEK-ERK1/2 pathway, and in turn, promotes carcinogenesis [23, 24]. After being screened from 45 patients with colorectal carcinoma, Src activity is considered as an independent indicator of poor clinical prognosis in all stages of human colon carcinoma [25-27].

Both Src and TOPK are very important in colon cancer, and furthermore, there exist the Src consensus substrate motif, pY[A/G/S/T/E/D] in TOPK. Therefore, we hypothesize whether Src could phosphorylate TOPK directly in colon cancer. In this study, we found that Src phosphorylated TOPK directly *in vitro* and *in vivo*, increased the stability and activity of TOPK. The interaction between Src and TOPK promoted carcinogenesis in colon cancer. Thus, these findings may help to predict prognosis or develop new therapeutic strategies to colon cancer.

## RESULTS

### Src phosphorylates TOPK at Y74 and Y272 *in vitro*

**Figure 1:**
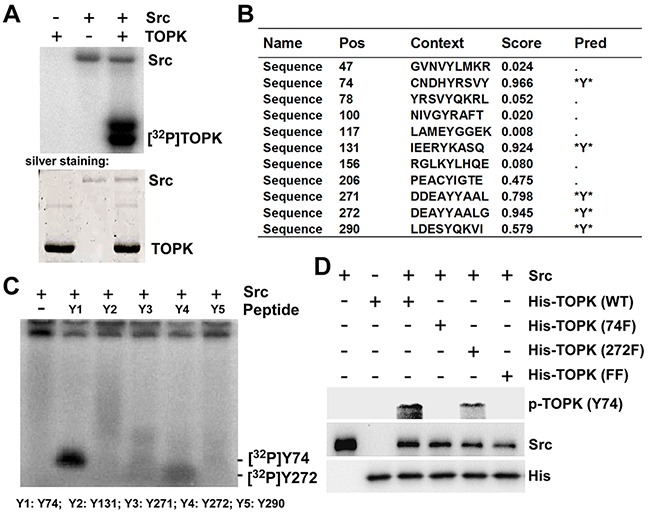
Src phosphorylates TOPK at Y74 and Y272 *in vitro* **A.** Active Src phosphorylated inactive TOPK *in vitro* in the presence of [γ-^32^P] ATP as visualized by autoradiograph. **B.** Potential phosphorylated tyrosine sites of TOPK were predicted by NetPhos 2.0 software program. **C.** Src phosphorylated TOPK at Y74 or Y272 in peptide mapping. Five synthesized peptides containing potential tyrosine sites were used as substrates in an *in vitro* kinase assay with active Src in the presence of [γ-^32^P] ATP and the results were visualized by autoradiography. **D.** Validation of anti phospho-TOPK (Y74) (p-TOPK (Y74)) in an *in vitro* kinase assay. Wild type His-TOPK (WT), single mutant His-TOPK (74F), single mutant His-TOPK (272F) or double mutant His-TOPK (FF) as shown was used as substrate for active Src. Reactive products were resolved by SDS-PAGE and visualized by Western blot with p-TOPK (Y74).

Both TOPK and Src play an important role in carcinogenesis and clinical prognosis in CRC, and there is a consensus phosphorylation site (Y272) of Src in TOPK. Therefore, we hypothesized that Src could phosphorylate TOPK. To test this idea, an *in vitro* kinase assay was performed in the presence of [γ-^32^P] ATP with Src as an active kinase and TOPK as a substrate. The data indicated that Src could phosphorylate TOPK *in vitro* (Figure 1A). The potential tyrosine phosphorylation sites of TOPK were predicted by NetPhos 2.0 (Figure 1B) [28]. Five high-score peptides were then designed and synthesized commercially (Y1-Y5) (PEPTIDE 2.0, Houston, TX, USA). The peptides were individually incubated with active Src in the presence of [γ-^32^P] ATP in an *in vitro* kinase assay. The results showed that both Y74 and Y272 were phosphorylated by Src, and the phosphorylation signal was even stronger at the site of Y74 (Figure 1C: Y1, Y4). To further confirm the results from peptide mapping, the antibodies recognizing phosphorylatedTOPK (phospho-TOPK (Y74) (p-TOPK (Y74)) or phospho-TOPK (Y272) (p-TOPK (Y272)) were prepared as described in Materials and Methods, but the p-TOPK (Y272) antibody failed to detect phospho-TOPK. The wild type (His-TOPK) (WT), Y74F TOPK (74F), Y272F TOPK (272F) and Y74Y272FF TOPK (His-TOPK) (FF) were purified from E coli respectively, and used as substrates for active Src in an *in vitro* kinase assay. The results of Western Blot using prepared p-TOPK (Y74) antibody showed that Src could phosphorylate TOPK at Y74, and the signal disappeared in the single mutant TOPK-74F and the double mutant TOPK-FF (Figure 1D). These data suggested that Src did phosphorylate TOPK at Y74 *in vitro*.

### Src binds with TOPK and phosphorylates TOPK at Y74 *ex vivo*

**Figure 2:**
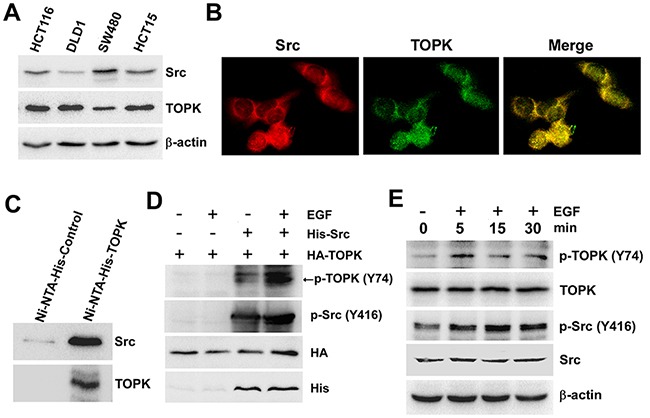
Src binds with TOPK and phosphorylates TOPK at Y74 *ex vivo* **A.** The levels of Src and TOPK in four colon cancer cell lines were shown. **B.** Colocalization of Src and TOPK was visualized by confocal microscope in SW480 cells. Cytoplasmic and nuclear staining of Src and TOPK was mostly merged together. **C.** Ni-NTA-His-TOPK bound with endogenous Src of SW480 cells. **D.** Src promoted phosphorylation of TOPK in 293T cells induced by EGF after co-transfected with pcDNA4-His-Src and pcDNA3-HA-TOPK (EGF 80 ng/ml; 30 min). **E.** EGF induced a time-dependent phosphorylation of TOPK at Y74 in SW480 cells (EGF 80 ng/ml; 0 min, 5 min, 15 min, 30 min).

To verify whether Src can phosphorylate TOPK at Y74 *ex vivo,* we firstly detected the expression of Src and TOPK in four kinds of different colon cancer cell lines. The results showed that the expression of Src was the highest in SW480 cells but the expression of TOPK was lowest (Figure 2A). Then we checked that if Src and TOPK could co-localize in SW480 cells under the confocal microscope. The result showed that Src (red) co-localized with TOPK (green) in both the cytoplasm and nucleus of SW480 cells (Figure 2B). Subsequently, the SW480 cells were harvested and lysed, and Ni-NTA-His-TOPK was used to pull down endogenous Src, and then Src was probed with anti-Src by Western blot. The results indicated that Src could directly bind with TOPK (Figure 2C). Next, we cotransfected pcDNA3-HA-TOPK and pcDNA4-His-Src into HEK293T cells, and the phosphorylation of TOPK at Y74 was analyzed by p-TOPK (Y74). The result indicated that the phosphorylation level of TOPK at Y74 was increased (lane 3) compared to the level of controls (lane 1 and 2) when cotransfected with Src and TOPK, and the level was dramatically increased (lane 4) after being stimulated by EGF (Figure 2D). Moreover, the phosphorylation level of TOPK at Y74 in SW480 cells at 0, 5, 15 or 30 minutes after EGF treatment was tested. The result showed that endogenous phosphorylation of TOPK at Y74 was increased after EGF treatment (Figure 2E). These data indicated that phosphorylation of TOPK at Y74 could be detected *ex vivo*.

### The phosphorylation of TOPK at Y74 was inhibited in colon cancer cells expressing low levels of Src

**Figure 3:**
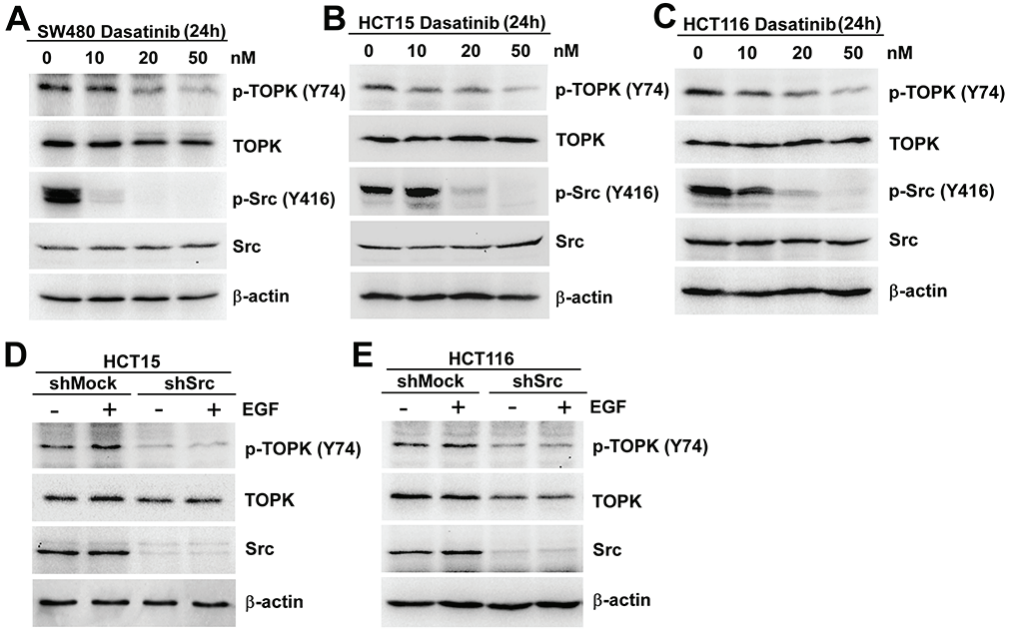
The phosphorylation of TOPK at Y74 was inhibited in colon cancer cells expressing low levels of Src SW480 **A.** HCT15 **B.** and HCT116 **C.** cells were treated with Dasatinib for 24 h in a dose-dependent manner and the samples were resolved by SDS-PAGE and analyzed by Western blot respectively. The stable shMock and shSrc in HCT15 **D.** or HCT116 **E.** cells were treated with EGF (80 ng/ml; 30 min), and analyzed by Western blot, respectively. Data are representatives of results from triplicate experiments.

Since Src could phosphorylate TOPK at Y74 *in vitro* (Figure 1) and *ex vivo* (Figure 2), what we explored next was if the phosphorylation of TOPK at Y74 was inhibited in colon cancer cells expressing low levels of Src. At first, the Src inhibitor, Dasatinib, was used to test this idea further. Dasatinib is known as a targeted therapeutic small-molecule Src inhibitor [29, 30]. We analyzed the endogenous phosphorylation level of TOPK at Y74 in three different colon cancer cell lines treated with Dasatinib. The results suggested that endogenous phosphorylation level of TOPK at Y74 was gradually decreased in a dose-dependent manner, and p-Src (Y416) was lowered after dasatinib treatment (Figure 3A, 3B and 3C). We then confirmed this idea in shMock- and shSrc-expressing cells. The results suggested that endogenous phosphorylation level of TOPK at Y74 was decreased in shSrc-expressing cells (Figure 3D and 3E).

All the first three figures supported the idea that TOPK could be phosphorylated by Src at Y74 *in vitro* and *ex vivo*, which may suggest a novel activation mechanism of TOPK.

### The phosphorylation of TOPK at Y74 and Y272 by Src promotes carcinogenesis *ex vivo*

**Figure 4:**
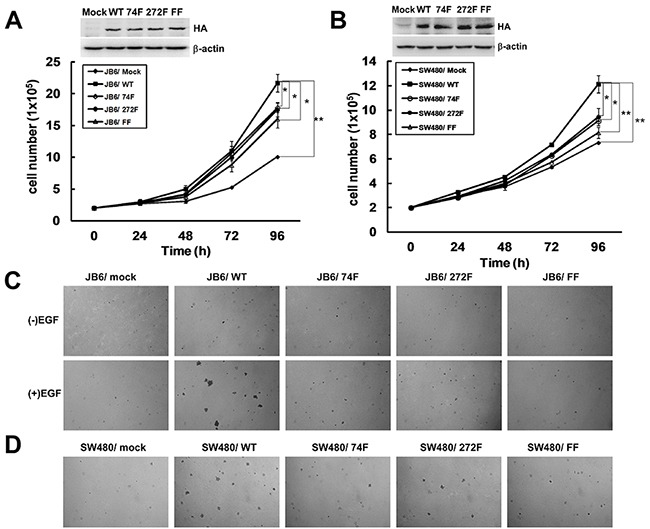
The phosphorylation of TOPK at Y74 and Y272 by Src promotes carcinogenesis *ex vivo* **A.** Growth curves of JB6 cells stably expressing pcDNA3-Mock (JB6/ Mock), pcDNA3-TOPK-WT (JB6/ WT), pcDNA3-TOPK-74F (JB6/ 74F), pcDNA3-TOPK-272F (JB6/ 272F), or double-mutant pcDNA3-TOPK-FF (JB6/ FF). Inset (top) showed verification of the cell lines identified by Western blot. Data are represented as means±SD of triplicate experiments. *, significantly (*P* < 0.05) decrease in cell number in JB6/ 74F, JB6/ 272F or JB6/ FF cells compared with JB6/ WT cells respectively. **, significantly (*P* < 0.01) increase in cell number in JB6/ WT cells compared with JB6/ Mock cells. **B.** Growth curves of SW480 cells stably expressing pcDNA3-mock (SW480/ Mock), pcDNA3-TOPK-WT (SW480/ WT), pcDNA3-TOPK-74F (SW480/ 74F), pcDNA3-TOPK-272F (SW480/ 272F), or double-mutant pcDNA3-TOPK-FF (SW480/ FF). Inset (top) showed verification of the cell lines identified by Western blot. Data are represented as means±SD of triplicate experiments. *, respective (*P* < 0.05) decrease in cell number in SW480/ 74F or 272F compared with SW480/ WT cells. **, significantly (*P* < 0.01) decrease in cell number in SW480/ FF cells compared with SW480/ WT cells and significantly (*P* < 0.01) increase in cell number in SW480/ WT cells compared with SW480/ Mock cells. **C.** Transfectants of JB6/ Mock, WT, 74F, 272F, or FF were compared for EGF-induced colony formation in soft agar. **D.** Transfectants of SW480/ Mock, WT, 74F, 272F, or FF were compared for colony formation in soft agar.

Next, the function of phosphorylation of TOPK at Y74, Y272, or Y74Y272 by Src was tested respectively. As reported before, TOPK could promote EGF-induced cell transformation and colon cancer development [7]. Thus we constructed TOPK mutants Y74F, Y272F or Y74Y272FF and set up stable cell lines in JB6 and SW480 cell lines in which the expression of endogenous TOPK was low. Growth curves of JB6/ Mock and different JB6/ TOPK cell lines were compared and the results showed that the growth of JB6/ WT cells was remarkably faster than that of JB6/ Mock cells. The growth of JB6/ 74F, 272F, or FF cells were slower than that of JB6/ WT cells.

(Figure 4A). The similar results were observed in SW480 stable cell lines (Figure 4B). Next, the anchorage-independent colony formation ability of stable cell lines was tested. Compared with JB6/ WT group, the colonies formed by JB6/ 74F, JB6/ 272F and JB6/ FF cells were significantly fewer and smaller (Figure 4C). Consistent with the data in the previous study, [7] the colonies formed by JB6/ WT cells were significantly more in number and larger in size than that formed by JB6/ Mock cells (Figure 4C). Similarly, the colonies formed by SW480/ 74F, SW480/ 272F and SW480/ FF cells were also relatively fewer and smaller than those formed by SW480/ WT cells (Figure 4D). All above data showed that mutated TOPK at Y74F, Y272F, or Y74Y272FF could block the anchorage-independent growth ability *ex vivo,* suggesting that the phosphorylation of TOPK by Src at Y74 and Y272 could promote carcinogenesis *ex vivo*. TOPK double mutant Y74Y272FF was used in the following experiment since either Y74F or Y272F had function.

### The phosphorylation of TOPK at Y74, Y272 by Src enhances the activity of TOPK

**Figure 5:**
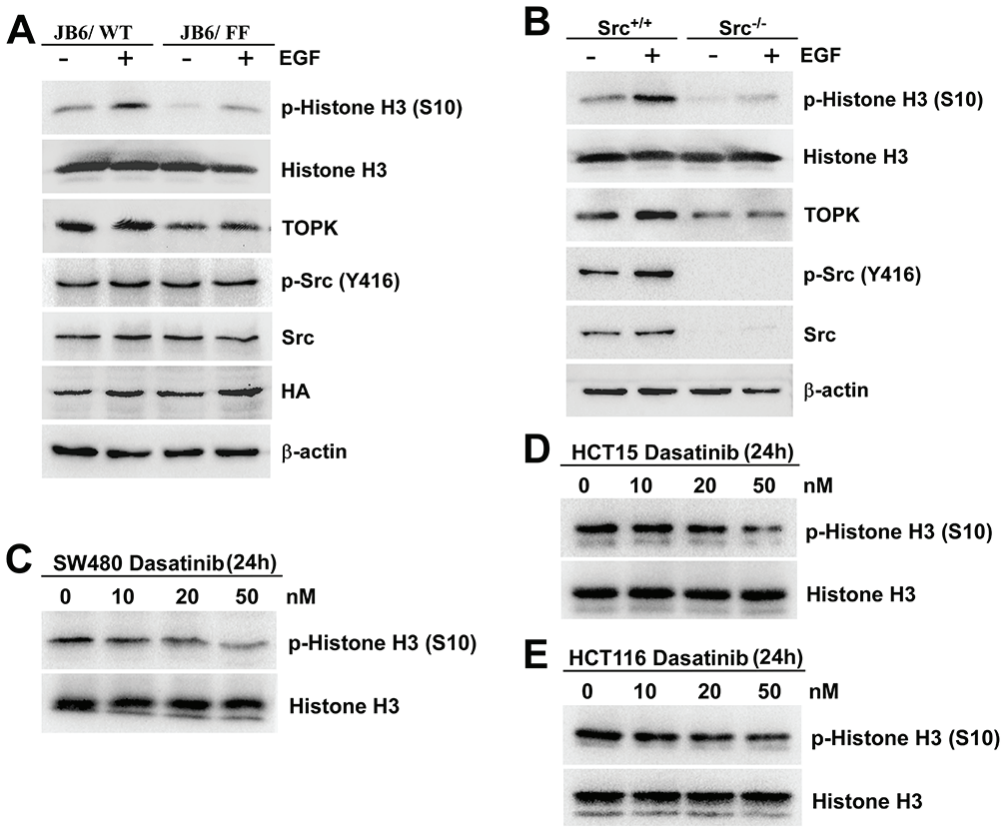
The phosphorylation of TOPK at Y74, Y272 by Src enhances the activity of TOPK **A.** JB6/ WT and JB6/ FF cells were treated with EGF (20 ng/ml; 15 min), and the samples were analyzed by Western blot. **B.** Src^+/+^ and Src^−/−^ MEFs were treated with EGF (20 ng/ml; 15 min), and analyzed by Western blot. SW480 **C.** HCT15 **D.** and HCT116 **E.** cells were treated with Dasatinib for 24 h in a dose-dependent manner. Then Histones were isolated, resolved by SDS-PAGE and analyzed by Western blot, respectively. Data are representatives of results from triplicate experiments.

Since the phosphorylated TOPK by Src promoted cell transformation, the next question that attracted us was whether the activity of TOPK was enhanced. The phosphorylated Histone H3 at Ser10 is the most widely established and used mitotic marker [31, 32], and is one of TOPK's substrates confirmed by J. H. Park et al [14]. The results showed that the level of phospho-Histone H3 (p-Histone H3 (S10)) dramatically decreased in JB6/ FF cells (Figure 5A), suggesting the decreasing activity of TOPK with double-mutation. Similar result was observed in Src knockout (Src^−/−^) cells (Figure 5B). Meanwhile, the levels of p-Histone H3 (S10) also decreased with 50nM dasatinib treatment in the three colon cancer cells (Figure 5C, 5D and 5E). These data implied that the phosphorylation of TOPK at Y74, Y272 by Src enhanced the activity of TOPK.

### The phosphorylation of TOPK by Src enhances the stability of TOPK

**Figure 6:**
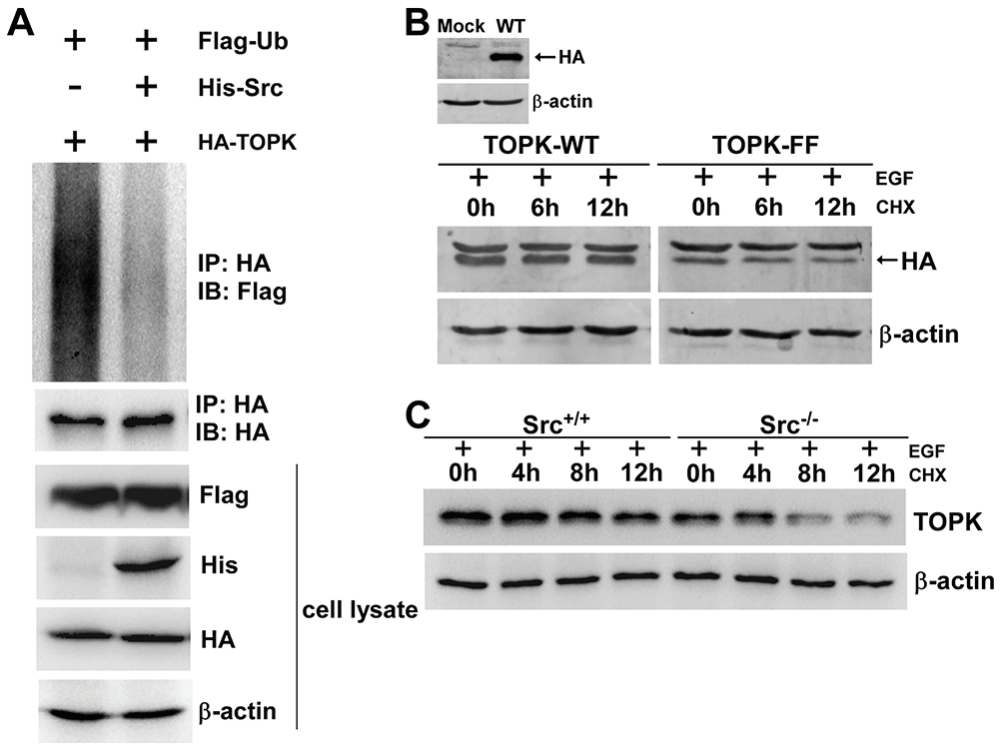
The phosphorylation of TOPK by Src enhances the stability of TOPK **A.** Phosphorylation of TOPK by Src prevented TOPK ubiquitination. HEK293T cells were transfected with pCMV-Flag-ubiquitin (Flag-Ub), pcDNA3-HA-TOPK (HA-TOPK), and pcDNA4-His-Src (His-Src) as indicated. The cells were harvested 48 h after transfection. Then the samples were immunoprecipitated (IP) with anti-HA and detected with anti-Flag by Western blot. The transfection efficiency and equal protein loading were verified by Western blot using the whole cell lysate. **B.** HEK293T cells were transfected with pcDNA3-HA-TOPK wild type (TOPK-WT) and pcDNA3-HA-TOPK double mutant (TOPK-FF), and then were treated with EGF (80 ng/ml; 30 min) followed by addition of CHX (100 μg/ml) to prevent new protein synthesis. The time-dependent stability of TOPK was detected with anti-HA by Western blot. **C.** Src^+/+^ and Src^−/−^ MEFs were treated with EGF (20 ng/ml; 15 min) followed by addition of CHX (100 μg/ml) to prevent new protein synthesis. Lysates were collected at the indicated time points. The protein level of TOPK was detected by Western blot.

As shown in Figure 5B and Figure 3D and 3E, we found that the level of TOPK was obviously decreased in Src^−/−^ cells and shSrc-expressing cells. This attracted us to explore whether the phosphorylation of TOPK by Src could affect the stability of TOPK. First, His-Src, Flag-ubiquitin or HA-TOPK were cotransfected into HEK293T cells. 48 hours later, cell extracts from each group were immunoprecipitated with anti-HA, and Flag-ubiquitin was detected by Western blot. The results indicated that the overexpression of Src dramatically reduced the ubiquitination level of TOPK compared with the control (Figure 6A). Next, to further illuminate these result, we examined the half-life of TOPK. pcDNA3-HA-TOPK wild type (TOPK-WT) and pcDNA3-HA-TOPK double mutant (TOPK-FF) were transiently transfected into HEK293T cells. Then the cells were treated with EGF for 30 min followed by treatment with CHX to assess the stability of TOPK at various time points. The result showed that TOPK-FF had shorter half-life than that of TOPK-WT (Figure 6B). Subsequently, the half-life of endogenous TOPK in Src^+/+^ cells was also compared with Src^−/−^ cells. The results indicated that the half-life of TOPK in Src^+/+^ cells was much longer than that in Src^−/−^ cells (Figure 6C). In summary, these results elucidate that phosphorylation of TOPK by Src enhances the stability of TOPK.

### Src phosphorylates TOPK to promote tumorigenesis of colon cancer *in vivo*

**Figure 7:**
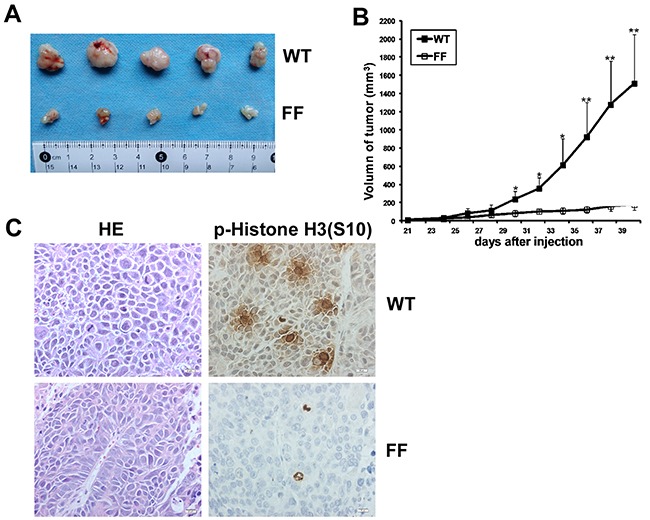
Src phosphorylates TOPK to promote the tumorigenesis of colon cancer *in vivo* **A.** Tumors dissected from SW480/ WT or SW480/ FF group were shown. **B.** Tumor growth curve from mice injected with SW480/ WT or SW480/ FF cells. Data are expressed as means±SE of 5 mice in each group. The asterisk indicated a significant increase in tumor size in SW480/ WT injected mice compared with SW480/ FF injected mice. *P < 0.05, **P < 0.01. **C.** Left, H&E stained tumor sections from mice injected with either SW480/ WT or SW480/ FF cells. Poorly differentiated adenocarcinoma cell clusters were found in the two groups. Right, IHC analysis of p-Histone H3 (S10) expression in tumor sections from mice injected with either SW480/ WT or SW480/ FF cells. Nuclear expression of p-Histone H3 (S10) was detected in the tumor sections from SW480/ WT cells, while very weak staining of p-Histone H3 (S10) was detected from SW480/ FF cells. Magnification, 400×.

Based on the *ex vivo* data, we then verified the above results *in vivo*. We compared the ability of SW480/ WT cells to form tumors in athymic nude mice with that of SW480/ FF cells. SW480/ WT or SW480/ FF cells (3×10^6^) were injected subcutaneously into the right flank of athymic Balb/c mice. 5 out of the 8 mice injected with both experimental cells developed tumors. The tumors from mice injected with SW480/ WT cells grew from 0 mm^3^ to nearly 1000 mm^3^ within 5 weeks. And the tumors from SW480/ FF cells inoculated mice were much smaller (Figure 7A). Tumor growth curves indicated that the increasing growth rate in the mice injected with SW480/ FF cells was significantly lower than that in the mice inoculated with SW480/ WT cells (Figure 7B). After the tumors of mice injected with SW480/ WT cells grew to 1000 mm^3^, the mice were euthanized and the tumors were dissected and then sent for hematoxylin & eosin (H&E) staining and immunohistochemical (IHC) analysis. The results showed a solid arrangement of tumor cells with high nuclear/cytoplasmic ratio, marked nuclei pleomorphism, visible mitosis, and numerous blood vessels in some areas (Figure 7C left panel). It suggested both of them were poorly differentiated adenocarcinoma cells. IHC analysis of the tumor samples from two groups of mice showed that p-Histone H3 (S10) was more highly expressed in SW480/ WT cells than that in SW480/ FF cells (Figure 7C right panel). Therefore, the tumorigenic properties of SW480/ FF cells significantly reduced, suggesting that Y74 and Y272 were important sites for TOPK function *in vivo*.

## DISCUSSION

Several groups of scientists have reported that TOPK can regulate cellular proliferation and promote tumorigenesis. Furthermore, TOPK is considered to be an unfavorable prognostic indicator in colon cancer patients and this provides the opportunity to predict prognosis in these patients using anti-TOPK [19]. However, non-phospho-TOPK antibody can not fully represent the activity of TOPK, and the phosphorylation TOPK at T9 can not promote tumorigenesis (Supplementary Figure S1). There must exist other activation mechanisms of TOPK related to tumorigenesis. So far it is only reported that hDlg [2], P53 [33], E2F-CREB/ATF [34], c-Myc-E2F1 [35], Cdk1 [2, 3, 36], p38 and ERK2 [7] could interact with TOPK and regulate TOPK at the transcriptional or posttranscriptional level. Among them, ERK2, p38 or Cdk1 can phosphorylate TOPK at T9 and the positive feedback loop between TOPK and ERK2 seemed to contribute to transformation in colon cancer [7]. In this study, Src was identified to be a novel upstream kinase of TOPK *in vitro* and *ex vivo*. The phosphorylation of TOPK at Y74, Y272 by Src can promote tumorigenesis of colon cancer cells *ex vivo* and *in vivo* and activate TOPK–Histone H3 pathway.

Colon cancer patients could benefit both from the detection of the disease at early stages and from the advance of targeted therapy [37, 38]. In clinic, it is reported previously that Src activity gradually increases with the progression from benign polyps to primary colonic lesions to liver metastatic cancer [24, 27]. TOPK is highly expressed in CRC compared with the matched normal colorectal tissue [7]. In this study, the activity of TOPK was first time reported dramatically increased after phosphorylated by Src at Y74 and Y272. The phosphorylation of TOPK by Src at Y74 and Y272 were closely related to tumorigenic. We generated p-TOPK (Y74) and p-TOPK (Y272) antibodies in this study, and only p-TOPK (Y74) could be used *in vitro* and *ex vivo*. Both antibodies failed to detect in IHC study. More specific phosphorylated TOPK (Y74) and (Y272) antibodies are needed urgently to be used in clinicopathological study in colon cancer, because accurate understanding TOPK activity in colon cancer patients will provide more valuable information.

Our study showed TOPK could be degraded through ubiquitination pathway, the phosphorylation of TOPK by Src inhibited this process, and the half-life of TOPK dramatically decreased in Src^−/−^ MEFs compared with Src^+/+^ MEFs. It has been reported that FBW7 can bind with ERK [39] or c-Jun [40], and TOPK can bind with ERK [7] or c-Jun [41] as well. Therefore, FBW7 may be related to the ubiquitination of TOPK.

In addition to the above, our results may bring benefits to the option of potential double-target for novel colon cancer targeted therapy. Although some single-agent Src inhibitors have been applied in clinical trials, preclinical studies hinted that a combined therapy might greatly increase the efficiency of Src inhibitors in colon cancer [42]. At the same time, TOPK gradually becomes an attractive molecular target for the treatment of a wide range of human cancers. At present, Caffeic acid (CaA) [43], HI-032 [44] and OTS964 [45] are the reported inhibitors for TOPK, and OTS964 is considered to be the best one for its high bioavailability. Coincidentally, the kinase profile analysis in that research indicated that OTS964 inhibited not only 79.7% of TOPK activity, but also 87.6% of Src activity [45]. In our study, the activity and stability of TOPK were increased by Src, and therefore the inhibition of both Src and TOPK, like OTS964, will be very efficient to cure colon cancer. It is promising to develop more drugs targeting both TOPK and Src for anticancer therapy.

In conclusion, our study elucidates that Src is a novel upstream kinase of TOPK, the phosphorylation of TOPK at Y74, Y272 by Src increases TOPK stability and promotes tumorigenesis of colon cancer. The specific phosphorylated TOPK antibodies would be made to predict prognosis for colon cancer patients instead of non-phospho-TOPK antibody. And it may develop new therapeutic strategies to colon cancer.

## MATERIALS AND METHODS

### Antibodies and reagents

TOPK and β-actin were purchased from Santa Cruz Technology, Inc (Santa Cruz, CA, USA). Phospho-Src (Tyr416) (D49G4) Rabbit mAb, Src (32G6) Rabbit mAb, Histone H3 (D1H2) XP Rabbit mAb, phospho-Histone H3 (Ser10) (D2C8) XP Rabbit mAb, and HA-Tag Rabbit polyclonal antibody were purchased from Cell Signaling Technology, Inc (Boston, MA, USA). Ubiquitin Rabbit polyclonal antibody was purchased from Proteintech Group, Inc (Chicago, USA). His-Tag Mouse monoclonal antibody (4E6) and HA-Tag Mouse monoclonal antibody (1B10) were obtained from Pregene, Inc (Beijing, China). HRP-labeled Goat anti Mouse IgG (H+L) and Goat anti Rabbit IgG (H+L) were purchased from EarthOx, LLC (San Francisco, CA, USA). Donkey anti-Rabbit IgG (H+L) Alexa Fluor^®^ 546 Red and Donkey anti-Mouse IgG (H+L) Alexa Fluor^®^ 488 Green were purchased from Invitrogen, Inc (Carlsbad, CA, USA). Phospho-TOPK at Y74 or Y272 antibodies were prepared by Abgent, Inc (Suzhou, China). All antibodies were used following the instructions of the respective manufacturers. The small hairpin RNA constructs against Src (5′-GACAGACCTGTCCTTCAAGAA-3′) used in this study was from the BioMedical Genomics Center at the University of Minnesota. Dasatinib was purchased from Selleckchem, Inc (Houston, TX, USA).

### Cell culture, plasmids and transfection

JB6 Cl41, HEK293T, SW480, HCT116 and HCT15 cells were purchased from American Type Culture Collection (ATCC). Cells were cultured following the procedures provided by ATCC and were used within 6 months of resuscitation. Src wild-type (Src^+/+^) and knockout (Src^−/−^) mouse embryonic fibroblasts (MEFs) were gifts from Imamoto A (University of Chicago, Chicago, IL 60637, USA). The cells were cultured with Dubelco's minimum essential medium (DMEM) supplemented with 10% fetal bovine serum (FBS). TOPK mutants at Y74, Y272, or Y74Y272 (designated Y74F, Y272F, and FF) were performed with the QuikChange Mutagenesis Kit (Stratagene, Inc., La Jolla, CA, USA). The mutant plasmids were sent to Sangon Biotech, Inc. (Shanghai, China) for DNA sequencing.

The transfect reagent, Simple-Fect, was purchased from Signaling Dawn Biotech (Wuhan, China). G418 (Sigma, St. Louis, USA) was used to set up stable cell lines. The pcDNA3-HA-TOPK, pcDNA4-His-Src and vector plasmids were transfected using Simple-fect into 293T cells at 50- 60% confluence following the manufacturer's suggested protocol.

### Bacterial expression and purification of the His-TOPK

PET-His-TOPK-WT and pET-His-TOPK-mutants were expressed in *E. coli* BL21 bacteria. Bacteria were grown at 37°C to an absorbance of 0.6–0.8 at 600 nm, induced with 1 mM isopropyl β-D-thiogalactopyranoside (IPTG) at 30°C for 4h. All proteins were purified using nickel-nitrilotriacetic acid agarose (Qiagen, Inc., Valencia, CA, USA) overnight at 4°C and eluted with 200 mM imidazole. After protein quantitation, the samples were separated by 10% SDS polyacrylamide gel for electrophoresis (SDS-PAGE), and visualized by Coomassie Brilliant Blue staining.

### *In vitro* kinase assay

The Src active kinase and 10×kinase buffer were purchased from Millipore Corp. (Billerica, MA, USA). The inactive substrate (2 μg) and the active kinase (0.2 μg in a 30 μl reaction) were incubated at 32°C for 40 min in 1×kinase buffer containing 100 μmol/L unlabeled ATP or 1 μCi [γ-^32^P] ATP. The samples were added with 5×SDS buffer and then resolved by SDS-PAGE and visualized by autoradiography or Western blot.

### Confocal laser scanning fluorescence microscopy

SW480 cells were fixed in methanol (−20°C) and blocked in 5% normal goat serum at room temperature for 1 h. Then the cells were incubated overnight with the primary antibodies to detect TOPK and Src at 4°C. On the second day, the cells were incubated for 1 h at room temperature with the Alexa Fluor 546 (red for Src) or Alexa Fluor 488 (green for TOPK) conjugated secondary antibody while being protected from light. Colocalization of proteins was observed by laser scanning confocal microscopy (NIKON C1^si^ Confocal Spectral Imaging System, NIKON Instruments Co., Japan). 5% normal goat serum instead of the primary antibodies was used as a negative control.

### Western blot and immunoprecipitation

Cells (8×10^5^) were cultured in 10 cm diameter dishes to 70-80% confluence, starved for 24 hours and treated with EGF ( R&D lot: HLM481304). After that the cells were harvested and disrupted in 300 μl of RIPA buffer (1X PBS, 1% Nonidet P-40, 0.5% sodium deoxycholate, 0.1% SDS, 1mmol/L Na_3_VO_4_, and 1 mmol/L aprotinin and 1 mmol/L phenylmethylsulfonyl fluoride). The samples were sonicated 15 seconds for three times and centrifuged at 13,000 rpm for 10 minutes. Histones were isolated as described previously [10]. Then the quantity of protein was determined by the Bradford method [46]. The samples (30-50 μg protein) with 5×SDS loading buffer were heated at 95°C for 10 minutes, and then cooled on ice. After that, the samples were separated on a 10%-15% SDS-PAGE and subsequently transferred onto a PVDF membrane (Millipore, Billerica, MA, USA). Then antibody-bound proteins were detected by chemiluminescence (BIO-RAD, USA). The untreated cell samples were used as negative controls. The samples for immunoprecipitation were harvested in 1% CHAPS instead of RIPA buffer. Equal amounts of protein (1–2mg) were subjected to immunoprecipitation following the manufacturer's suggested protocol. (http://www.scbt.com/protocols.html?protocol=immunoprecipitation).

### Growth curve analysis

Cells were plated at 2 × 10^5^ cells per 10 cm dish and counted in triplicate at different time points using a hemacytometer for generating a growth curve.

### Anchorage-independent cell transformation assay

Different cell lines (8 × 10^3^/well) in a 6-well plate were exposed or not exposed to EGF (20 ng/ml) and cultured in 1ml of 0.33% BME (Eagle basal medium, Sigma-Aldrich Corp.) agar (Sigma-Aldrich Corp.) containing 10% FBS over 3ml of 0.5% BME agar containing 10% FBS. The cells were maintained in a 37°C, 5% CO_2_ incubator for 5-10 days and then their colonies were observed by microscopy.

### Tumor xenografts

Athymic Balb/c nude mice (4–6-week-old males) were purchased from Beijing HFK Bioscience CO., LTD (Beijing, China). The mice were randomized into two groups. Each of the different cell lines (3×10^6^ in 200 μl PBS) was injected subcutaneously into the right flank of the athymic Balb/c nude mice, and the tumors were measured every other day. We estimated the tumor volume (V) from their length (l), width (w), and height (h) using the following formula: V = 0.52 (l×w×h). The mice were monitored until total volume of the tumors reached 1000 mm^3^. The tumors were dissected and sent for IHC analysis at the Department of Pathology in Xijing Hospital. The animal experiments were performed following the protocols approved by the Laboratory Animal Center of the Fourth Military Medical University.

### Immunohistochemistry staining for samples from node mice

After the mice were euthanized, the tumors were fixed in 4% formalin, routinely processed, and embedded in paraffin. Sections of 5 μm were placed on glass slides for H&E staining and IHC analysis. All the samples from node mice were diagnosed by the Department of Pathology of Xijing Hospital. The tumor sections were stained with the p-Histone H3 (S10) antibody (1:200). Images were obtained and 400× magnified using an Olympus Imaging System Microscope.

### Cycloheximide treatment

HEK293T Cells which were transiently transfected pcDNA3-HA-TOPK (WT) and pcDNA3-HA-TOPK (FF) plasmids as before were treated for 15 minutes with EGF, and then the medium was supplemented with 100 μg/ml of cycloheximide (CHX; Sigma, St. Louis, USA) to block protein synthesis. The cells were collected at various time points and expression of TOPK was visualized by Western blot. Src^+/+^, Src^−/−^ MEFs were also carried out in the same way.

### Statistical analysis

Significant differences were determined by one-way analysis of variance (ANOVA). The values were presented as means ± SEM. All statistical tests were two sided, and *P* < 0.05 was considered significant (*P < 0.05, **P < 0.01).

## SUPPLEMENTARY FIGURE


