# Supplementary material for: Phosphorylation of TOPK at Y74, Y272 by Src increases the stability of TOPK and promotes tumorigenesis of colon
Source: Oncotarget. 2016 Mar 21;7(17):24483–94. doi: 10.18632/oncotarget.8231 (PMC5029716; doi:10.18632/oncotarget.8231)
Supplement: Supplementary file 1 [file oncotarget-07-24483-s001.pdf]

SUPPLEMENTARY FIGURE

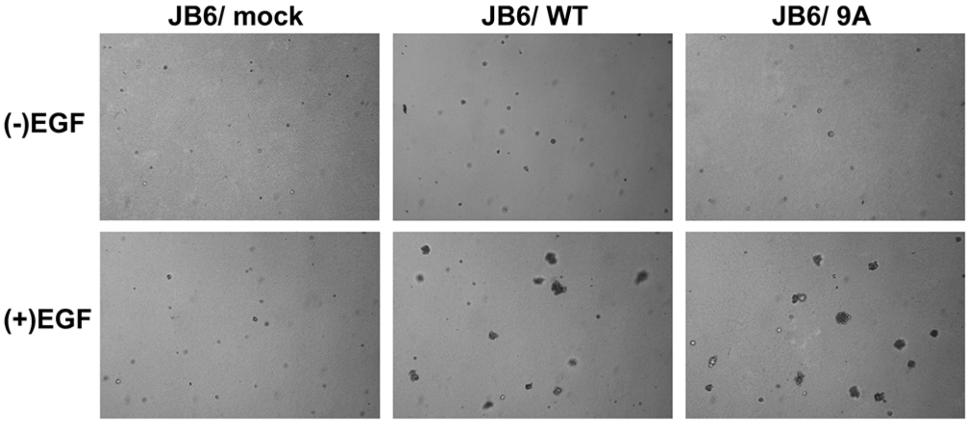

Supplementary Figure S1: The phosphorylation of TOPK at T9 can not promote tumorigenesis *ex vivo*. JB6 cells stably expressing pcDNA3-Mock (JB6/ Mock), pcDNA3-TOPK-WT (JB6/ WT), pcDNA3-TOPK-9A (JB6/ 9A) were set up. Then transfectants of JB6/ mock and JB6/ 9A were compared for EGF-induced colony formation in soft agar.
